# The Physiological Impact of GFLV Virus Infection on Grapevine Water Status: First Observations

**DOI:** 10.3390/plants11020161

**Published:** 2022-01-07

**Authors:** Anastazija Jež-Krebelj, Maja Rupnik-Cigoj, Marija Stele, Marko Chersicola, Maruša Pompe-Novak, Paolo Sivilotti

**Affiliations:** 1 School for Viticulture and Enology, University of Nova Gorica (UNG), Glavni trg 8, 5271 Nova Gorica, Slovenia; maja.cigoj1@gmail.com (M.R.-C.); Marusa.Pompe.Novak@nib.si (M.P.-N.); paolo.sivilotti@uniud.it (P.S.); 2 Department of Biotechnology and Systems Biology, National Institute of Biology (NIB), Večna Pot 111, 1000 Ljubljana, Slovenia; marija.stele@gmail.com (M.S.); marko.chersicola@gmail.com (M.C.); 3 Regional Development Agency of Northern Primorska Ltd. Nova Gorica (RRA SP), Trg Edvarda Kardelja 3, 5000 Nova Gorica, Slovenia; 4 Department of Fruit Growing, Viticulture and Oenology, Agricultural Institute of Slovenia (KIS), Hacquetova Ulica 17, 1000 Ljubljana, Slovenia; 5 Department of AgriFood, Environmental and Animal Sciences, University of Udine, Via Palladio 8, 33100 Udine, Italy

**Keywords:** grapevine, water status, virus infection, GFLV, xylem vessel occlusion, gene expression

## Abstract

In a vineyard, grapevines are simultaneously exposed to combinations of several abiotic (drought, extreme temperatures, salinity) and biotic stresses (phytoplasmas, viruses, bacteria). With climate change, the incidences of drought in vine growing regions are increased and the host range of pathogens with increased chances of virulent strain development has expanded. Therefore, we studied the impact of the combination of abiotic (drought) and biotic (*Grapevine fanleaf virus* (GFLV) infection) stress on physiological and molecular responses on the grapevine of cv. Schioppettino by studying the influence of drought and GFLV infection on plant water status of grapevines, on grapevine xylem vessel occlusion, and on expression patterns of 9-cis-epoxycarotenoid dioxygenase 1 (*NCED1*), 9-cis-epoxycarotenoid dioxygenase 2 (*NCED2*), *WRKY* encoding transcription factor (*WRKY54*) and *RD22*-like protein (*RD22*) genes in grapevines. A complex response of grapevine to the combination of drought and GFLV infection was shown, including priming in the case of grapevine water status, net effect in the case of area of occluded vessels in xylem, and different types of interaction of both stresses in the case of expression of four abscisic acid-related genes. Our results showed that mild (but not severe) water stress can be better sustained by GFLV infection rather than by healthy vines. GFLV proved to improve the resilience of the plants to water stress, which is an important outcome to cope with the challenges of global warming.

## 1. Introduction

Grapevines are exposed to several abiotic (drought, extreme temperatures, salinity) and biotic stresses (phytoplasmas, viruses, bacteria), especially as they are propagated in a vegetative way. Both abiotic and biotic stresses are responsible for dramatic economic losses and represent the main limiting factor for viticulture worldwide. Biotic stress can cause reduction of grapevine vigor, substantial crop losses [1,2] and often the decline of grapevines, which also affects the commercial value and lifetime of a vineyard. One of the most important and widespread virus diseases of grapevine is Grapevine fanleaf degeneration [3,4], which occurs in all grapevine-growing regions throughout the world [5]. This disease is caused by *Grapevine fanleaf virus* (GFLV), which is a member of the genus *Nepovirus* [6]. GFLV causes degeneration and malformations of leaves, shoots and clusters. It is also responsible for significant reduction of crop yields by up to 80%, and it affects the longevity of grapevines [5,7,8].

Abiotic stresses often affect grapevine water status, but the magnitude of influence also depends on soil and climate characteristics [9] as well as on grapevine health status. Grapevine water status can accurately be assessed by means of stem water potential (Ψ_STEM_) using the Scholander pressure chamber [10]. Well-watered grapevines report midday Ψ_STEM_ values above −0.6 MPa, while lower values represent conditions of mild (−0.9 < Ψ_STEM_ < −0.6 MPa), moderate (−1.1 < Ψ_STEM_ < −0.9 MPa), severe (−1.4 < Ψ_STEM_ < −1.1 MPa), and excessive water stress (Ψ_STEM_ < −1.4 MPa), respectively [10]. Root hydraulic conductance (RHC, nLH_2_Os^−1^m^−1^ MPa^−1^) has been used as a good parameter to describe modifications in water uptake from the root-soil interface to the apoplast of the leaves.

Plants in nature are simultaneously exposed to combinations of biotic and abiotic stresses. There can be either no direct interaction between the multiple simultaneous stresses (only net effect) or multiple simultaneous stresses can result in enhanced susceptibility or enhanced tolerance. Besides, sequential individual stresses may lead to priming (enhanced tolerance) or predisposition (weakened plant defenses) to subsequent stresses [11]. Biotic and abiotic signaling pathways may share multiple nodes, and their output may have significant overlap for plants to survive under complex environmental conditions [12,13,14]. A consequence of environmental and genetic interactions through a complex network that implies physiological, molecular and biochemical responses, results in adaption to abiotic or biotic stress. The adaption to biotic stresses, involves the development of specific molecular mechanisms in plants to detect pathogens and pests and to activate defense responses. In parallel, it has been found that pathogens have developed sophisticated molecular mechanisms to deregulate the biosynthesis of hormones and/or to interfere with hormonal signaling pathways, thus facilitating the overcoming of plant defense mechanisms [15,16]. Drought-tolerant plants have developed strategies to cope with and adapt to abiotic and on the other hand also to biotic stresses. Moreover, viruses can even extend survival of their hosts under conditions of abiotic stress that could benefit hosts. Inoculation with four different RNA viruses, *Brome mosaic virus* (BMV), *Cucumber mosaic virus* (CMV), *Tobacco mosaic virus* (TMV) and *Tobacco rattle virus* (TRV), improved plant tolerance to abiotic stress, as the onset of drought symptoms in virus-infected plants was delayed in comparison to the mock-inoculated plants [17].

Physiological responses of plants to abiotic or biotic stress can result also in vessel occlusion in xylem vascular bundles. Biotic and abiotic stresses have been reported to cause vessel occlusion, including freezing, drought, mechanical wounding (including pruning), flooding, insect attack or pathogen infection. Vessel occlusion caused by bacterial pathogens has been extensively studied, while there were only few reports of vessel occlusion by viruses. For example, stem sections from plants of Indian mustard (*Brassica juncea*) infected with Turnip mosaic virus (TuMV) developing systemic hypersensitive resistance had a significantly higher percentage (63%) of occluded xylem vessels than those from plants developing systemic mosaic (27%), or mock-inoculated plants (9 to 11%) [18]. Vessel occlusion can be due to the formation of tyloses or to the deposition of gums/gels. Vessel occlusion can happen either naturally with xylem aging of heartwood, or in sapwood both normally or in response to various stresses [19,20,21,22]. At present, it is commonly accepted that embolism precedes vessel occlusion [23], although vessel embolism is not required for wound-induced tylosis development in grapevine [24]. Cavitation, as a prerequisite for occlusion, could explain more frequent tylosis formation in large vessels that are more prone to embolism than narrow ones [25,26,27]. Embolism-related tylosis formation is considered as a quick response to stressful conditions and explains why tyloses are frequent in vessels close to wounds or around sites of pathogen inoculation preventing the spread of pathogens, embolism and in reducing water loss in non-functional xylem [19]. Most plants have developed a mechanism to restore vessel functionality by refilling embolized vessels. In grapevine, vessel refilling was shown to be dependent on water influx from surrounding living tissue [23]. Tylosis formation or gel/gum depositions might have a role in embolism repair by contributing to osmotic-related mechanisms for refilling of embolised vessels [19].

Plant growth, development and the responses of plants to drought and virus infection are regulated by plant hormones [28]. Abscisic acid (ABA) is the main drought-induced hormone that regulates the expression of many genes related to drought responses [29,30,31]. ABA biosynthesis is largely induced through transcriptional factors, although regulation of the specific activities of ABA biosynthesis enzymes also exists. Those regulators are induced by distinct stresses, among them, the most widely studied are abiotic stresses (drought and salinity). Stress tolerance mechanisms are controlled by a variety of genes, which are expressed at different growth stages. An assortment of genes with diverse functions are induced or repressed by plant stress, such as genes encoding enzymes regulating biosynthesis of a variety of sugars, transcription factors and regulatory factors [32,33,34,35]. Therefore, a fine-tune regulation is necessary because phytohormone pathways are linked to each other in the complex and obscure network in which all contribute to hormone balance. On the other hand, virus-induced biotic stresses have been more intensely investigated in the lats fewt years [36,37,38,39,40,41,42,43], although the molecular mechanisms by which grapevines response to GFLV infection are still not fully understood. 

In this study, we studied the impact of the combination of abiotic (drought) and biotic (GFLV infection) stresses on physiological and molecular responses on the grapevine of cv. Schioppettino. A complex response of grapevine to the combination of drought and GFLV infection was shown; including priming in the case of grapevine water status, net effect in the case of area of occluded vessels in xylem, and different types of interaction of both stresses in the case of expression of four ABA-related genes.

## 2. Results

### 2.1. The Influence of Drought and GFLV Infection on Plant Water Status of Grapevines

**Figure 1:**
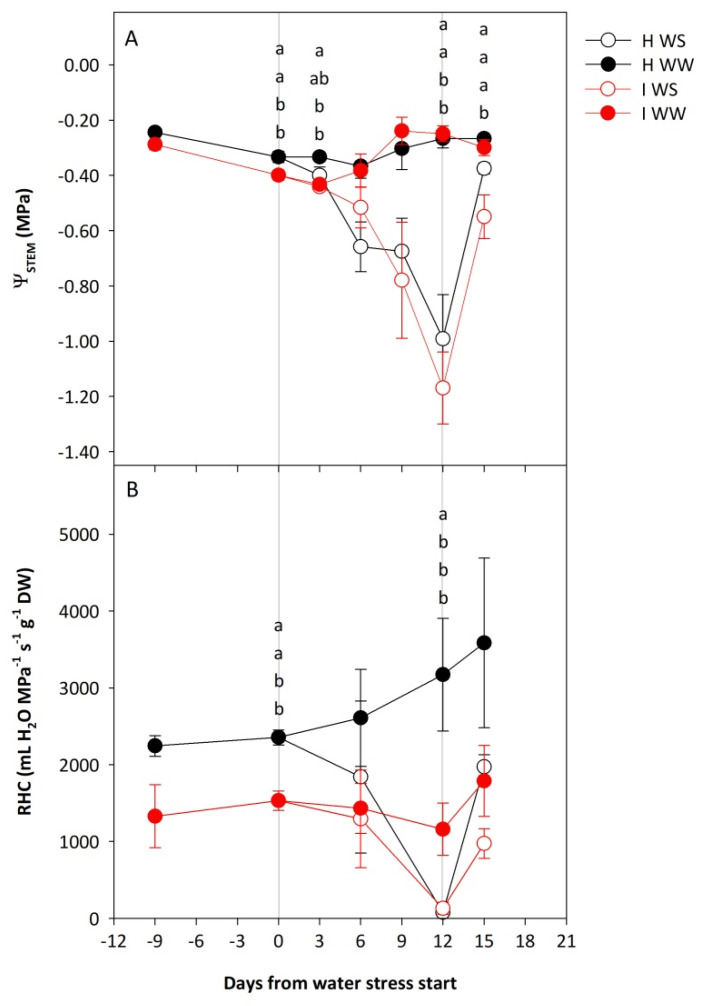
Stem water potential (Ψ_STEM_; **A**) and root hydraulic conductivity (RHC; **B**) of potted own-rooted grapevines of cv. Schioppettino before the water stress, during the water stress, and after the recovery. Plants are separated regarding to plant health and water status: well-watered healthy (H WW), well-watered GFLV-infected (I WW), water-stressed healthy (H WS) and water-stressed GFLV-infected (I WS) plants. Error bars represent standard errors. For each time point separately, data were processed through ANOVA (significance reported in Supplementary Table S1), and means were separated with a Student Newman Keuls test (*p* < 0.05; different letters represent significant differences between means). Grey lines represent the beginning and the end of water stress period.

The study of the stem water potential (Ψ_STEM_) and root hydraulic conductivity (RHC) in the potted own-rooted grapevines of cv. Schioppettino (*Vitis vinifera* L.) revealed a complex interaction of GFLV infection and drought stresses that led to an impact on the water status of plants. The measurements collected before the water stress start highlighted the impact of GFLV infection alone, significant one day before the start of water stress (Figure 1); both Ψ_STEM_ and RHC were lower in the case of infected well-watered (I WW) vines, and the magnitude of difference was much higher in the case of RHC (Figure 1B). The imposition of water stress promoted a reduction of both parameters, and at 12 days after water depletion, both healthy water-stressed (H WS) and infected water-stressed (I WS) vines showed significantly lower values of Ψ_STEM_ as compared to WW vines (Figure 1A). The same trend was also ascertained for RHC, but only healthy well-watered (H WW) vines reported significantly higher values of such a parameter as compared to all the other treatments under comparison (Figure 1B). A priming effect was observed in the combination of both stresses; GFLV-infected plants namely showed a trend for being more resistant to mild water stress than healthy plants, as the drops of their Ψ_STEM_ and RCH were lower at 6 days after a different water regime as compared to healthy plants (Figure 1). On the other hand, at severe water stress 12 days after different water regimes, the average Ψ_STEM_ value of GFLV-infected plants was lower than the average Ψ_STEM_ value of healthy plants. At severe water stress 12 days after a different water regime, the RCH of both healthy and infected plants dropped close to zero. After re-watering, Ψ_STEM_ of H WS plants recovered back to the values of WW vines, while the values of I WS vines remained still significantly lower as compared to the other treatments. As regard RHC, water replacement allowed a partial increase of this parameter in WS treatments, but the highest values were ascertained again in the case of H WW, even if this was not statistically proved.

### 2.2. The Influence of Drought and GFLV Infection on Grapevine Xylem Vessel Occlusion 

**Figure 2:**
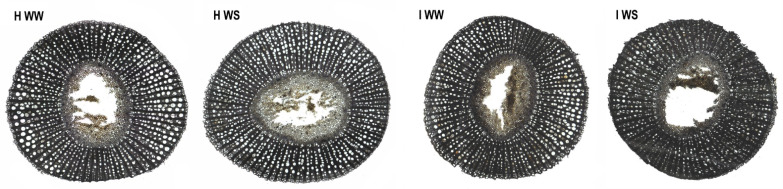
Cross-sections through the canes of well-watered healthy (**H WW**), well-watered GFLV-infected (**I WW**), water-stressed healthy (**H WS**) and water-stressed GFLV-infected (**I WS**) potted own-rooted grapevines of cv. Schioppettino 6 days after the start of different water regime obtained by light microscope.

The study of the stem tissue sections of own-rooted cv. Schioppettino included the measurements of the area, the diameter and the ratio between open and occluded vessels of the healthy and GFLV-infected vines exposed to drought and in well-watered conditions (Figure 2). The average vessel diameter in I WW before the start of water stress was slightly lower as compared to H WW canes (76 µm and 79 µm, respectively; *p* < 0.001). Consequently, the average vessel cross section area (3372 µm^2^ and 3607 µm^2^, respectively; *p* < 0.001) and the portion of the sums of vessel cross section areas in the whole xylem area (15% and 20%, respectively) were lower in I WW vines as compared to H WW.

**Figure 3:**
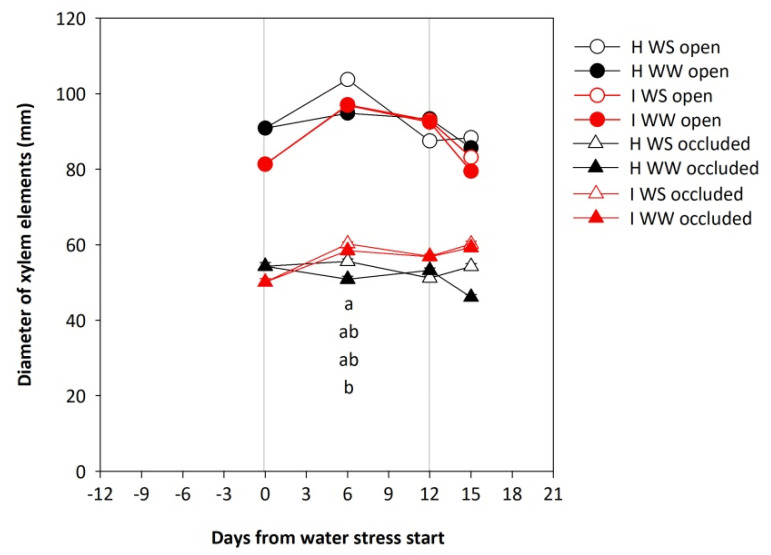
Diameter of xylem elements of potted own-rooted grapevines of cv. Schioppettino before the water stress, during the water stress, and after the recovery. Plants are separated regarding to plant health and water status: well-watered healthy (H WW), well-watered GFLV-infected (I WW), water-stressed healthy (H WS) and water-stressed GFLV-infected (I WS) plants. Error bars represent standard errors. For each time point separately, data were processed through ANOVA (significance reported in Supplementary Table S1), and means were separated with a Student Newman Keuls test (*p* < 0.05; different letters represent significant differences between means). Grey lines represent the beginning and the end of water stress period.

The average diameter of open xylem elements was larger than the average diameter of occluded xylem elements. At the beginning of the experiment, before the imposition of water stress, the average diameter of both open and occluded vessels was tendentially lower in the case of GFLV-infected vines as compared to the healthy ones. Examining separately open and occluded vessels, no differences were observed between the four treatments under comparison, even if GFLV treatments reported slightly lower values of open and higher values of occluded vessel diameters at the time of recovery (16 days after water stress start; Figure 3).

**Figure 4:**
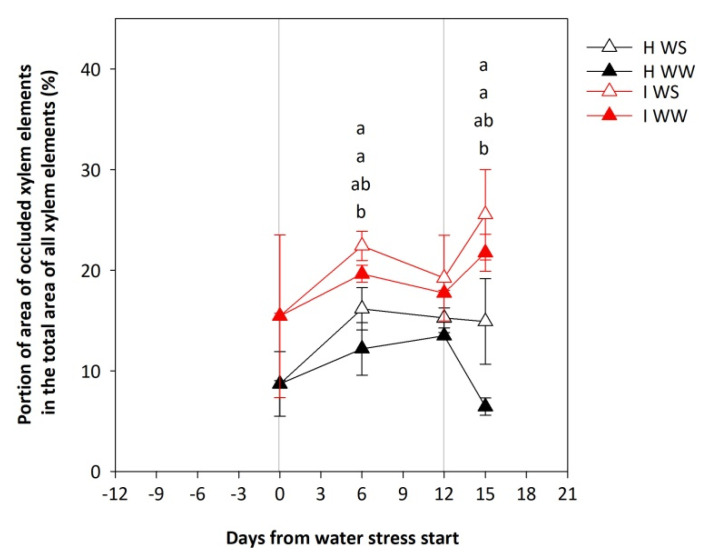
Portion of area of occluded xylem elements in the total area of all xylem elements of potted own-rooted grapevines of cv. Schioppettino before the water stress, during the water stress, and after the recovery. Plants are separated regarding their plant health and water status: well--watered healthy (H WW), well-watered GFLV-infected (I WW), water-stressed healthy (H WS) and water-stressed GFLV-infected (I WS) plants. Error bars represent standard errors. For each time point separately, data were processed through ANOVA (significance reported in Supplementary Table S1), and means were separated with a Student Newman Keuls test (*p* < 0.05; different letters represent significant differences between means). Grey lines represent the beginning and the end of water stress period.

Water stress promoted an increase in the portion of occluded vessels in both healthy and GFLV-infected vines as compared to well-watered vines (Figure 4). In H WW vines a significantly lower portion of occluded xylem elements were observed as compared to both I WW and H WS at 6 days after water stress start and after recovery (Figure 4).

**Figure 5:**
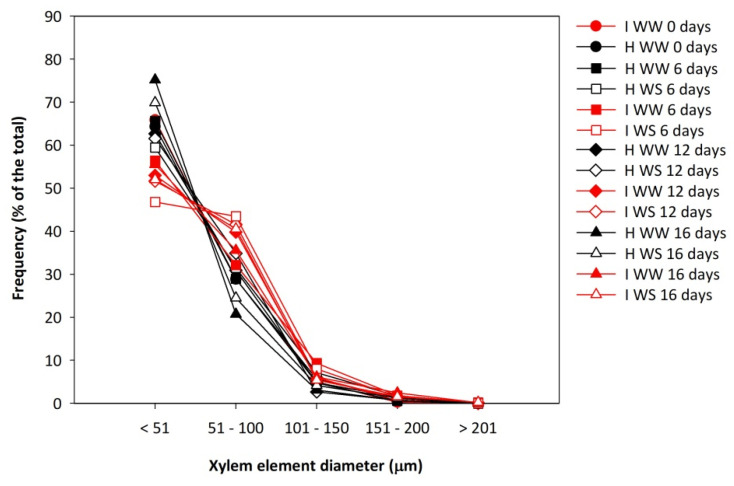
Relative frequency of occluded xylem elements of different diameter class of shoot sections of potted own–rooted grapevines of cv. Schioppettino before the water stress and during the water stress, and after the recovery. Plants are separated regarding to plant health, water status and days from water stress start: well–watered healthy (H WW), well–watered GFLV–infected (I WW), water–stressed healthy (H WS) and water–stressed GFLV–infected (I WS) plants.

Moreover, the occluded xylem elements were most frequently the ones with the smallest diameters up to 50 µm. In healthy vines, the frequency of occluded xylem elements with the smallest diameters of up to 50 µm was higher than in infected vines (*p* < 0.01), while in GFLV-infected vines, the frequency of occluded xylem elements with diameters ranging from 51–100 µm was higher than in healthy vines (*p* < 0.01) (Figure 5). The other xylem diameter classes were just slightly affected by GFLV. 

### 2.3. The Influence of Drought and GFLV Infection on Expression Patterns of NCED1, NCED2, RD22 and WRKY54 Genes in Grapevines 

In order to investigate the response of grapevine to a combination of GFLV infection and water stress on a molecular level, the expression of *NCED1, NCED2, RD22* and *WRKY54* genes was monitored in young leaves of grapevines of cv. Schioppettino, planted in pots.

**Figure 6:**
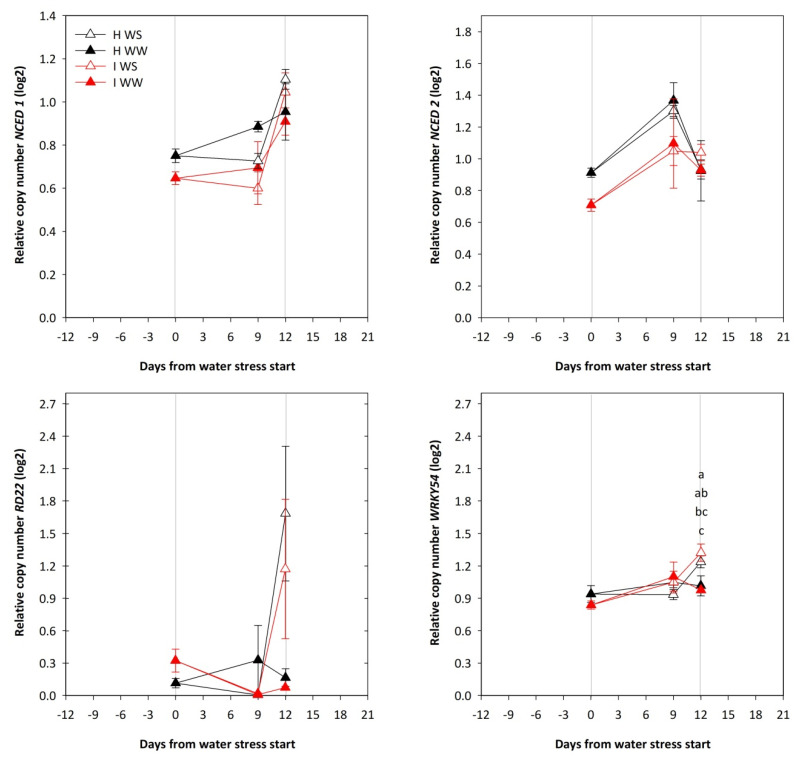
Expression of *NCED1*, *NCED2*, *RD22* and *WRKY54* genes in the young leaves of potted own-rooted grapevines of cv. Schioppettino before and during the water stress. Plants are separated regarding to plant health and water status: well-watered healthy (H WW), well-watered GFLV-infected (I WW), water-stressed healthy (H WS) and water-stressed GFLV-infected (I WS) plants. Error bars represent standard errors. For each time point separately, data were processed through ANOVA (significance reported in Supplementary Table S2), and means were separated with a Student Newman Keuls test (*p* < 0.05; different letters represent significant differences between means). Grey lines represent the beginning and the end of water stress period.

The expression of *NCED1* showed upregulation at 12 days of water depletion due to WS treatments while no difference in expression due to WS was found for *NCED2* (Figure 6). The same trend as for *NCED1* was observed also for *RD22* and *WRKY54*, and for the latter gene the difference in expression between well-watered and water-stressed vines was significant between GFLV-infected plants but not between healthy vines. Besides, the expression of *NCED1* and *NCED2* showed a trend of downregulation due to GFLV infection.

## 3. Discussion

In the context of climate change, the incidences of drought in vine growing regions have increased [44], and there is evidence to suggest that climate change will expand the host range of pathogens and increase the chances of virulent strain development [45]. Therefore, it is becoming very important to understand the impact of a combination of increasing stresses on grapevines. In order to investigate the response of grapevine to the combination of biotic (GFLV infection) and abiotic (drought) stress, healthy and GFLV-infected self-rooted grapevines of cv. Schioppettino in pots in a greenhouse were exposed to two different water regimes: they either were exposed to water stress (drought) or were well-watered. We decided on a pot experiment in a greenhouse as it enabled a more controlled experiment than in a vineyard. To monitor the effect of drought on plant water status, two parameters were followed: stem water potential (Ψ_STEM_) and root hydraulic conductivity (RHC).

Before the start of water stress, in the well-watered conditions, RHC and Ψ_STEM_ were significantly lower in the case of GFLV-infected vines. Water stress significantly lowered RHC at 12 days from the start of water stress in both healthy and GFLV-infected vines. By comparing this experiment with another carried out in field conditions, there was a milder impact of GFLV infection on young self-rooted grapevines grown in pots in the greenhouse, as our previous studies showed a much lower Ψ_STEM_ in GFLV-infected vines of cv. Schioppettino which was statistically significant as compared to healthy controls [36]. A milder impact of GFLV infection on young self-rooted grapevines was proven also by the absence of any visible symptoms of GFLV infection.

In the first days after the start of water stress, the drop in RHC and Ψ_STEM_ was greater in healthy vines compared with GFLV-infected water-stressed vines (Figure 1). This indicates that priming grapevines with GFLV infection increased the tolerance of grapevines to mild water stress. An indication that priming grapevines with GFLV infection increases the tolerance of grapevines to mild water stress could be found also in the previous study of cv. Refošk in vineyard conditions [36]. We could speculate that the reduction of the number of open vessels could act as a primer reducing Ψ_STEM_ because of the limited transpiration. Plenty of literature is available on the effects of water stress on grapevine physiology, while virus infection has only slightly been examined. In one of these articles, the authors [46] highlighted the effects of water stress and GLRaV infection in an experiment similar to the one presented here. Virus infection resulted in a significantly diminished transpiration because of the reduction of petiole hydraulic conductance. In our experiment, when water stress became more intense, the priming effect of GFLV virus infection was overcome, and Ψ_STEM_ lowered down more intensively as compared to healthy vines.

RHC and Ψ_STEM_ are indicators of hydraulic conductance of sap through root and shoot sap pathways [47,48]. Breakage of water columns caused by embolism and/or occlusion drastically reduces the hydraulic conductance [49,50]. A reduction of RHC and Ψ_STEM_ can be a consequence of low soil water content or embolism and/or occlusion of the vessels, which cannot be refilled during the night period [51]. In our study, an obvious inverse proportion between RHC/Ψ_STEM_ values and the portion of occluded xylem vessels was shown, as in healthy well-watered vines with the highest RHC/Ψ_STEM_ values and the lowest portion of occluded xylem vessels was observed; conversely, in GFLV-infected water-stressed vines with the lowest RHC/Ψ_STEM_ values, the highest portion of occluded xylem vessels was observed, indicating that xylem vessel occlusions might be the reason for RHC/Ψ_STEM_ reduction.

Sun et al. [52] showed that occlusions of xylem vessels in the grapevine are formed by tyloses and gum/gel deposits. Occlusions of xylem vessels in the grapevine were caused by bacteria *Xylella fastidiosia* (the causing agent of Pierce’s disease) [52] and fungi *Phaeomoniella chlamydospora* [53] were extensively studied, while there were only a few reports of vessel occlusion by viruses [18]. It was shown that some fungi cause occlusion only when the plant was simultaneously exposed to infection and abiotic stress, such as drought [54].

Wheeler et al. [55] reported that the incidence of tyloses and gum/gel deposits increased from 4% to 25% and from 11% to 24% of the taxa, respectively, with the increase of taxa vessel diameter from very narrow vessel taxa (<50 μm), via narrow (50–100 μm) and wide (100–200 μm), to very wide vessel taxa (>200 μm). Sun et al. [52] did not see obvious differences in tylose-forming capacity between vessels of different diameters in the grapevine. On the contrary, Pouzoulet et al. [56] showed that the vessels with the smaller diameter became occluded faster after pruning and the vessels with the larger diameter needed more time to reach complete occlusion. Similarly, an inverse proportion of vessel diameter to occlusion in the grapevine was found in our present study. Namely, in healthy well-watered grapevines, 64% of very narrow vessels (<50 μm), 18% of narrow vessels (50–100 μm), 6% of wide vessels (100–200 μm) and only 2% of very wide vessels (>200 μm) were occluded. Both GFLV infection and drought increased the number of occluded vessels. When the grapevines were exposed to the both stresses simultaneously, their effect was synergistical, resulting in occlusion of 71% of very narrow vessels (<50 μm), 39% of narrow vessels (50–100 μm), 13% of wide vessels (100–200 μm) and 4% of very wide vessels (>200 μm).

Lovisolo and Schubert [50] reported that the vessel transectional areas in water-stressed grapevines of cv. Freisa grafted on Kober 5BB in glasshouse in pots were lower than in irrigated plants. The vessel diameters most frequently ranged between 60 and 80 μm for both irrigated and water-stressed plants; however, diameters larger than 80 μm were more frequent in irrigated plants. As a consequence, the average vessel transectional area was about 35% lower in water-stressed rather than irrigated plants [50]. In our experiment on self-rooted grapevines of cv. Schioppettino, the vessel transectional area and total transectional area of vessels were reduced by GFLV infection, but not by water stress.

Although not significant, the expression of the 9-*cis*-epoxycarotenoid dioxygenase 1 and 2 (*NCED1* and *NCED2*) genes, related to water stress, were proven to be downregulated in GFLV-infected vines during the first period of water stress. When water stress became stronger, the differential expression of water-stress related genes in GFLV-infected vines resulted in a more intense reduction of Ψ_STEM_ as compared to healthy vines. The heavier water stress conditions at 12 days after the water stress start promoted an upregulation of the *NCED1* gene, ABA-responsive gene (*RD22*) and a gene involved in the ABA responsive signaling network (*WRKY54*). The responses of grapevines to drought are often associated with an accumulation of ABA in the petiole xylem and leaves [57,58], and consequently with stomatal closure [58] and decreased plant hydraulic conductance [59,60]. Additionally, it was shown that a decrease in the leaf water potential might enhance stomatal sensitivity to ABA [58,61]. This indicates that the chemical signals are important players in plant adaption to water stress. The role of *NCEDs*, the key genes in the ABA biosynthetic pathway, in the drought stress, by which the ABA biosynthesis pathway is regulated, was proposed by Wan et al. [62]. A positive correlation was found between the upregulation of *NCED* mRNA and the increased amount of NCED protein during the course of water deficit stress. Furthermore, ABA levels increased due to the overexpression of *NCED*. Expression studies have indicated that the regulation of *NCED1* [63] gene expression in leaves (but not in roots) is associated with the amount of ABA in the xylem sap. This observation was supported also by an examination of gene expression in leaves and roots from a shade house experiment [64]. Surprisingly, in the present study, the expression of *NCED2*, a gene involved in ABA biosynthesis, was not affected in leaves by the water deficit. 

This study has provided some important outlines for a better understanding of the effects of GFLV infection on the grapevine responses to drought conditions. GFLV-infected vines showed a lower value of RHC in well-watered conditions due to their smaller vessel diameter, but mainly to an increased percentage of occluded vessels. The reduction of water transport represents a positive characteristic of a vigorous variety such as cv. Schioppettino; the reduced supply of water limits canopy development and therefore plant transpiration, making plants more resistant to a mild and moderate water shortage. We showed that mild water stress can be better sustained by GFLV infection than by healthy vines. With the clonal selection, the grapevine varieties are virus-free, but in many cases they are more sensitive to abiotic stresses and to the variability of meteorological conditions between seasons. In the case of cv. Schioppettino, GFLV was proven to improve the resilience of the plants to water stress, an important outcome to cope with the challenges of global warming.

## 4. Materials and Methods

### 4.1. Plant Material

To study the impact of GFLV on the water status of grapevines under the controlled conditions of the greenhouse, 1-year-old shoots of grapevines (*Vitis vinifera* L.) of cv. Schioppettino were collected in the vineyard in Prepotto in winter, from six healthy and seven GFLV-infected grapevines. 

The samples collected in vineyard were analysed by DAS-ELISA (Bioreba kits) for the presence of Grapevine fanleaf virus (GFLV), Arabis mosaic virus (ArMV), Grapevine leafroll associated virus (GLRaV)-1, -2, -3, -4–-9, Grapevine virus A (GVA), and Grapevine fleck virus (GFkV), Tomato black ring virus (TBRV), Grapevine chrome mosaic virus (GCMV) (Agritest test), Tomato ringspot virus (ToRSV), Raspberry ringspot virus (RpRSV), Strawberry latent ringspot virus (SLRSV) and Tobacco ringspot virus (TRSV) and by DASI-ELISA for the presence of GVB (Grapevine virus B). Optical density (OD) was measured after 30 min, 1 h, 2 h and 18 h of incubation with substrate (1 mg/mL of paranitrophenyl phosphate) at 405 nm using a plate reader (Tecan SunriseTM, Männedorf, Switzerland). Data were processed using MagellanTM data analysis software. Elisa reads were considered positive when they reached values higher than 2-fold of the value of the negative controls. Samples that were negative for all tested viruses were considered as healthy, and samples there that were negative for all tested viruses except GFLV were considered to be GFLV-infected.

Collected healthy and GFLV naturally infected shoots were stored at 4 °C until they were propagated in a greenhouse. 2-buds shoot cuttings were planted in 1 m × 1 m plates filled with vermiculite and maintained in a growing chamber at 25 °C until the roots were formed. Thereafter, sixty self-rooted grapevines were planted into 3 L pots filled with Agriperlite BPB Vic and Goldhumus pflanzverde (3:1, *v*/*v*), and maintained in a greenhouse. Before the experiment, the plants were separated in to 4 groups of 15 plants relating to plant health and water status: well-watered healthy plants (H WW), well-watered GFLV-infected plants (I WW), water-stressed healthy plants (H WS) and water-stressed GFLV-infected plants (I WS). Well-watered plants were regularly irrigated to keep their stem water potential (Ψ_STEM_) between −0.2 MPa and −0.6 MPa. Water-stressed plants were left to dehydrate until their Ψ_STEM_ dropped down to −1.3 ± 0.1 MPa. Afterwards, they were recovered by a water supply. Irrigation scheduling was managed in a precise manner by daily weighing of the pots before (BW) and after watering (AW) at 10:00 a.m.; the difference [AW_day n_ and BW_day n+1_] was supplied to restore the water lost by evapotranspiration (daily irrigation is reported in Supplemental Table S3).

The environmental conditions of the greenhouse were monitored during the trial period (from 15 June to 13 July 2021) using iButton sensors/mini-data loggers (SPR Hygrochron temperature/humidity logger iButton with 8 kB data-log memory, Maxim Integrated, San Jose, CA, USA), collecting and storing the data on an hourly basis (daily min/med/max temperature and relative humidity data are reported in Supplemental Table S3).

### 4.2. Stem Water Potential (Ψ_STEM_) and Root Hydraulic Conductivity (RHC) Measurements

The Ψ_STEM_ was measured 9 days before and 0, 3, 6, 9, 12 and 16 days after applying different water regime on 3 plants from each treatment under comparison. To measure the Ψ_STEM_, a leaf was covered with an aluminum-foil-covered plastic bag 1 h before the measurement. Afterwards, the leaves were excessed using a razor blade, and Ψ_STEM_ rapidly assessed using a Scholander pressure chamber (Soil Moisture Co., Santa Barbara, CA, USA).

The root hydraulic conductivity (RHC) was measured 9 days before and 0, 6, 12 and 16 days after applying a different water regime on 3 plants from each treatment with the pressure-flux technique [65]. The stem of a grapevine with intact roots (that were still in the soil) was cut and inserted into the upper hole of a pressure chamber filled with water. A pressure of 0.3 MPa was gradually applied inside the chamber and the sap at the top of the cut stem was collected three times for 3 min. Afterwards, the vines were up-rooted, the roots were washed, dried for 48 h in an oven at 105 °C and the dry weight of roots was weighted. The RHC was calculated as the amount of sap (nl) per pressure in the chamber (MPa) per time (s) per dry weight of roots (g).

### 4.3. Microscopy of Stem Sections

From the same plants that were used for RCH measurements 0, 6, 12 and 16 days after applying a different water regime, 1 cm long pieces of stem were cut and put in FFA fixative (formaldehyde: ethanol: acetic acid: water = 4%: 50%: 5%: 41%), 30%, 50% and 70% ethanol, for one week in each of the solutions. Afterwards, the samples were stored in 70% ethanol at 4 °C until cutting by microtome (Reichert-Jung) into 35 μm thick slices. Before cutting, the bark was removed. Three to ten minutes before cutting and occasionally between cutting, samples were hydrogenated by dipping into distilled water and dropping the distilled water on the sample, respectively. Slices were transferred by tweezers or a thin brush into a drop of distilled water on a microscope slide and covered with a cover slide. The specimens were examined using Axioscope 2 MOT (Zeiss) and imaged by AxioCam MRc (Zeiss) camera. By Axiovision Real 4.8 software partial images over the entire area of the slices were taken in the way that the edges of the adjacent partial images overlapped each other. Partial images were imported into the Fiji-win32 software and assembled into full images. Setting two different thresholds, converting images to masks, and the addition and subtraction of the masks enabled us to distinguish between open and occluded xylem vessels. By performing particle analysis (Analyze Particles), data on area (Area, Area Fraction), circumference (Perimeter) and diameter (Ferets Diameter) were obtained for individual open and occluded xylem vessels.

Altogether, 55,620 vessels (from 750 to 2211 per full image) were identified by particle analysis with Fiji-win32.exe. The data obtained were imported from Fiji-win32.exe into Microsoft Excel, where we calculated the average diameters and areas of individual open and occluded xylem vessels, the total surface area occupied by the open and occluded lumen of the xylem and the proportions of occluded and solid xylem vessels which occupy them in relation to the whole lumen of the xylem or in relation to the entire piston of the rod for well-watered healthy plants (WW H), well-watered GFLV-infected plants (WW I), water stressed healthy plants (WS H) and water-stressed GFLV-infected plants (WS I) 0, 6, 12 and 16 days after applying different water.

### 4.4. Gene Expression Analysis

To analyze gene expression, the third leaf from the top was collected 0, 9 and 12 days after applying different water regimes from 3 plants from each well-watered group (WW H and WW I) and from 6 plants from each group of water-stressed plants (WS H and WS I) separately. The leaves were immediately frozen in liquid nitrogen and stored at −80 °C. The grapevine material was stored at −80 °C until it was used for RNA extraction.

For total RNA extraction, the grapevine leaves were ground to a fine powder in liquid nitrogen. Subsequently, 100 ± 20 mg was placed into tubes and RLC extraction buffer was added and then vortexed vigorously and incubated for 3 min at 56 °C, centrifuged for 30 s at 10,000× *g*. The supernatants obtained were used in the subsequent steps of RNA extraction, using the RNeasy Plant Mini kits (Qiagen, Chatsworth, CA, USA) according to the manufacturer’s instructions. The isolated RNA was quantified using a Nanodrop spectrophotometer (NanoDrop Technologies). Additionally, RNA quality and concentration was measured using an Agilent 2100 bio-analyser, using 2 μL of each sample with Agilent RNA 6000 Nano kits. 

The expression of four target and two reference genes was determined by qPCR. For 9-cis-epoxycarotenoid dioxygenase 1 (*NCED1*), 9-cis-epoxycarotenoid dioxygenase 2 (*NCED2*), RD22-like protein (*RD22*), WRKY encoding transcription factor (*WRKY54*) and ubiquitin-conjugating enzyme 28 (*UBI_CF*) genes, the SYBR^®^ Green chemistry was used, while for cytochrome oxidase (*COX*) gene, the TaqMan chemistry was used. Assay-related information is given in Supplemental Table S4 [66,67,68,69,70]. The qPCR was carried out in a LightCycler^®^ 480 instrument (Roche, Applied Systems, USA), in 384-well plate format using universal cycling conditions (2 min at 50 °C, 10 min at 95 °C followed by 40 cycles at 95 °C for 10 s and 60 °C for 1 min). Each qPCR reaction was performed in a final reaction volume of 5 µL, which contained 2 µL cDNA and 3 µL mastermix (SYBR^®^ Green or TaqMan), 300 nM of each primer for the SYBR Green chemistry, and 300 nM primers and 150 nM probes for the TaqMan chemistry. For the SYBR^®^ Green chemistry, the Power SYBR^®^ Green PCR Master Mix (Applied Biosystems, USA) was used. For the TaqMan chemistry, the TaqMan Universal PCR Master Mix (Applied Biosystems, USA) was used. For SYBR Green chemistry, the dissociation curve (95 °C for 15 s, 60 °C for 15 s and 95 °C for 15 s) was performed to verify the specificity of the products and primer dimers.

The initial data analysis was performed with the Roche LightCycler Software, and then the Cq values were exported to Excel files for further analysis. The relative quantification of the samples with the calibration curve was used. For each amplicon, the calibration curve was constructed. The relative expression ratio was calculated based on the efficiencies of amplification of each amplicon in each sample, where the slope represents ΔCq between 10-fold and 100-fold dilutions, and the differences of the normalised Cq values between each individual sample and the control sample. The Cq values were normalised to the geometric mean of the expression of the two reference genes (*COX* and *UBI_CF*). The validation of the stability of their expression was carried out using geNorm [71,72], which calculated the gene stability measure for both of the reference genes in a given set of samples.

### 4.5. Statistical Data Processing

Average values and standard errors were calculated for all measurements. Data were processed through one-way ANOVA (*p*) indicated in Supplementary Tables S1 and S2) keeping separate the dates. When the test was significant, the means were separated using a Student Newman Keuls test (*p* < 0.05). On graphs, the letters were not reported on dates where the ANOVA was not significant. Statistical analyses were performed with the software R version 4.0.5 (31 March 2021) [73] and the packages ‘agricolae’ version 13-5 and ‘dplyr’ version 1.0.5.

## Supplementary Materials

The following supporting information can be downloaded at https://www.mdpi.com/article/10.3390/plants11020161/s1: Supplemental Table S1: Significance of ANOVA in stem water potential (Ψ_STEM_), root hydraulic conductivity (RHC), and vessel parameters among the four treatments under comparison in the potted own-rooted grapevines of cv. Schioppettino. Supplemental Table S2: Significance of ANOVA in 9-cis-epoxycarotenoid dioxygenase 1 (*NCED1*), 9-cis-epoxycarotenoid dioxygenase 2 (*NCED2*), *WRKY* encoding transcription factor (*WRKY54*) and *RD22*-like protein (*RD22*), genes among the four treatments under comparison in the potted own-rooted grapevines of cv. Schioppettino. Supplemental Table S3: Daily irrigation applied to each vine from the beginning to the end of the experiment of the well-watered (WW) and water-stressed (WS) plants, and environmental conditions monitored in the greenhouse during the course of the experiment. Supplemental Table S4: Primers and probes used for gene expression analysis.
