# Supplementary material for: The Physiological Impact of GFLV Virus Infection on Grapevine Water Status: First Observations
Source: Plants (Basel). 2022 Jan 7;11(2):161. doi: 10.3390/plants11020161 (PMC8780503; doi:10.3390/plants11020161)
Supplement: Supplementary file 1 [file plants-11-00161-s001.zip › plants-1544226-supplementary.pdf]

plants

MDPI

Supplementary Materials

The Physiological Impact of GFLV Virus Infection on Grapevine Water Status

Anastazija Jež-Krebelj[1,2,3,4,*], Maja Rupnik-Cigoj[1,2,3], Marija Stele[2], Marko Chersicola[2], Maruša Pompe-Novak[1,2] and Paolo Sivilotti[1,5]

[1] School for Viticulture and Enology, University of Nova Gorica (UNG), Glavni trg 8, 5271 Vipava, Slovenia;
[2] Department of Biotechnology and Systems Biology, National Institute of Biology (NIB), Večna Pot 111, 1000 Ljubljana, Slovenia
[3] Regional Development Agency of Northern Primorska Ltd. Nova Gorica (RRA SP), Trg Edvarda Kardelja 3, 5000 Nova Gorica, Slovenia
[4] Department of Fruit Growing, Viticulture and Oenology, Agricultural Institute of Slovenia (KIS), Hacquetova ulica 17, 1000 Ljubljana, Slovenia
[5] Department of AgriFood, Environmental and Animal Sciences, University of Udine, via Palladio 8, 33100 Udine, Italy
* Correspondence: anastazija.jezkrebelj@kis.si

Citation: Jež-Krebelj, A.; Rupnik-Cigoj, M.; Stele, M.; Chersicola, M.; Pompe-Novak, M.; Sivilotti, P. The Physiological Impact of GFLV Virus Infection on Grapevine Water Status: First Observations. *Plants* **2022**, *11*, 161. https://doi.org/10.3390/plants11020161

Academic Editor: Mario Ciaffi

Received: 22 December 2021
Accepted: 5 January 2022
Published: 7 January 2022

Publisher's Note: MDPI stays neutral with regard to jurisdictional claims in published maps and institutional affiliations.

Copyright: © 2022 by the authors. Licensee MDPI, Basel, Switzerland. This article is an open access article distributed under the terms and conditions of the Creative Commons Attribution (CC BY) license (https://creativecommons.org/licenses/by/4.0/).

Supplemental Table S1: Significance of ANOVA in stem water potential (Ψ_{STEM}), root hydraulic conductivity (RHC), and vessel parameters among the four treatments under comparison in the potted own-rooted grapevines of cv. Schioppettino.

**The days before/after the start of different water regime**	(Ψ_{STEM})	**RHC**	**diameter of open xylem**	**diameter of occluded xylem**	**Portion of area of occluded xylem elements in the total area of cross sections of all xylem elements (%)**
-9	ns	ns	---	---	---
0	*	**	ns	ns	ns
3	*	---	---	---	---
6	ns	ns	ns	*	*
9	ns	---	---	---	---
12	**	**	ns	ns	ns
15	***	ns	ns	ns	*

Supplemental Table S2: Significance of ANOVA in 9-cis-epoxycarotenoid dioxygenase 1 (*NCED1)*, 9-cis-epoxycarotenoid dioxygenase 2 (*NCED2*), *WRKY* encoding transcription factor (*WRKY54*) and *RD22*-like protein (*RD22*), genes among the four treatments under comparison in the potted own-rooted grapevines of cv. Schioppettino.

**The days before/after the start of different water regime**	***NCED1***	***NCED2***	***WRKY54***	***RD22***
0	ns	ns	ns	ns
9	ns	ns	ns	ns
12	ns	ns	ns	*

Supplemental Table S3: Daily irrigation applied to each vine from the beginning to the end of the experiment of the well-watered (WW) and water-stressed (WS) plants, and environmental conditions monitored in the greenhouse during the course of the experiment.

	Daily irrigation (L pot^{-1} d^{-1})		Daily temperature (°C)			
date	WW	WS	T min	T med	T max	Relative Humidity (%)
15/6	0.24	0.24	12.8	20.7	26.9	76
16/6	0.25	0.25	14.6	22.6	29.0	76
17/6	0.25	0.25	15.8	24.5	31.1	72
18/6	0.25	0.25	19.0	26.3	33.2	62
19/6	0.25	0.25	17.1	26.4	33.6	63
20/6	0.26	0.26	19.4	26.9	33.3	67
21/6	0.26	0.26	20.3	27.2	33.1	65
22/6	0.26	0.26	22.1	27.3	32.0	49
23/6	0.26	0.26	20.5	25.6	28.7	52
24/6	0.26	0.26	19.2	25.5	31.6	57
25/6	0.26	0.26	19.0	23.6	29.9	71
26/6	0.26	0.26	16.9	23.1	29.4	56
27/6	0.25	0	14.8	23.3	29.9	65
28/6	0.26	0	17.0	24.1	30.4	69
29/6	0.26	0	17.5	25.4	31.5	68
30/6	0.27	0	19.7	26.7	33.2	71
1/7	0.27	0	20.3	27.5	34.7	71
2/7	0.28	0	20.2	28.8	37.4	59
3/7	0.28	0	20.2	26.9	32.7	70
4/7	0.28	0	19.5	26.3	32.6	68
5/7	0.28	0	20.7	26.4	32.5	66
6/7	0.28	0	17.7	23.2	30.0	74
7/7	0.27	0	16.6	24.4	30.5	75
8/7	0.27	0	19.0	25.5	31.7	71
9/7	0.28	0	17.1	26.0	32.7	66
10/7	0.28	0.28	21.0	26.0	32.4	57
11/7	0.29	0.29	17.2	24.8	31.6	72
12/7	0.28	0.28	16.5	23.3	28.9	68
13/7	0.28	0.28	17.8	21.9	26.2	66

Supplemental Table S4: Primers and probes used for gene expression analysis.

Gene name	Target gene ID	Description	Forward primer sequence<br>Reverse primer sequence<br>Probe sequence	Source
NCED1	AY337613	9-cis-epoxycarotenoid dioxygenase 1	TCCCTCACGAGTTCCCTATG<br>ATATGCGGACCATCCCTCT	[66]
NCED2	AY337614	9-cis-epoxycarotenoid dioxygenase	GAATAGGGTTTTGGGGGAAC<br>GGAAGATCCAAGAGAGGGAAA	[66]
RD22	NM_001281183.1	RD22-like protein	CCCATCCTTGCTCTTATCTCT<br>CACCTCCAGGTTTTCCATTTC	[67]
WRKY54	Vv_10004898	WRKY encoding transcription factor	TCCCATATGAGAAAGGAAGAG<br>TCCGTCTACACCGCAGTC	[68]
COX	X83206	cytochrome oxidase	CGTCGCATTCCAGATTATCCA<br>CAACTACGGATATATAAGAGCCAAAACTG<br>FAM-TGCTTACGCTGGATGGAATGCCCT-TAMRA	[69]
UBI_CF	Vitvi19g00744	ubiquitin-conjugating enzyme 28	CTATATGCTCGCTGCTGACG<br>AAGCCAGGCAGAGACAACTC	[70]
